# Ability of Polyphosphate and Nucleic Acids to Trigger Blood Clotting: Some Observations and Caveats

**DOI:** 10.3389/fmed.2018.00107

**Published:** 2018-04-17

**Authors:** Stephanie A. Smith, Joshua M. Gajsiewicz, James H. Morrissey

**Affiliations:** ^1^Department of Biological Chemistry, University of Michigan Medical School, Ann Arbor, MI, United States; ^2^L.E.K. Consulting, Boston, MA, United States

**Keywords:** polyphosphate, nucleic acids, contact pathway, blood coagulation, DNA, RNA

## Abstract

Polyphosphate plays several roles in coagulation and inflammation, while extracellular DNA and RNA are implicated in thrombosis and as disease biomarkers. We sought to compare the procoagulant activities of polyphosphate versus DNA or RNA isolated from mammalian cells. In a recent study, we found that much of the procoagulant activity of DNA isolated from mammalian cells using Qiagen kits resisted digestion with nuclease or polyphosphatase, and even resisted boiling in acid. These kits employ spin columns packed with silica, which is highly procoagulant. Indeed, much of the apparent procoagulant activity of cellular DNA isolated with such kits was attributable to silica particles shed by the spin columns. Therefore, silica-based methods for isolating nucleic acids or polyphosphate from mammalian cells are not suitable for studying their procoagulant activities. We now report that polyphosphate readily co-purified with DNA and RNA using several popular isolation methods, including phenol/chloroform extraction. Thus, cell-derived nucleic acids are also subject to contamination with traces of cellular polyphosphate, which can be eliminated by alkaline phosphatase digestion. We further report that long-chain polyphosphate was orders of magnitude more potent than cell-derived DNA (purified *via* phenol/chloroform extraction) or RNA at triggering clotting. Additional experiments using RNA homopolymers found that polyG and polyI have procoagulant activity similar to polyphosphate, while polyA and polyC are not procoagulant. Thus, the procoagulant activity of RNA is rather highly dependent on base composition.

## Introduction

For years, *in vitro* studies of the contact pathway of blood clotting have relied on anionic substances like silica, kaolin, or diatomaceous earth to trigger this pathway. Although widely used in aPTT assays, none of these agents are likely physiologic or even pathophysiologic triggers of the contact pathway. Recent years have seen a renaissance in this pathway, linking it to thrombosis, inflammation, and innate immunity (1). This in turn has led to renewed interest in identifying the real physiologic or pathophysiologic activators of the contact pathway. Proposed candidates include polyphosphate (polyP) (2) and extracellular nucleic acids (3). In this article, we review recent studies that have investigated the procoagulant activities of polyP and nucleic acids, provide new data comparing their relative clotting activities, and point out some important caveats in interpreting the results of such studies.

Polyphosphate, widespread throughout biology, is a linear polymer of inorganic phosphates (4). Microorganisms are well known to accumulate polyP that is highly heterogeneous in length, ranging from just a few to over a thousand phosphates in length (4, 5). Various mammalian tissues are also reported to contain polyP of varying polymer lengths, including brain, heart, lung, bone, and prostate (6–9). In 2004, the Docampo laboratory reported that dense granules of human platelets contain abundant quantities of polyP, with a relatively narrow size distribution ranging from about 60 to 100 phosphates in length (10). PolyP, like other platelet granule contents, is secreted upon platelet activation. In 2006, we reported for the first time that polyP is a potent modulator of the human blood clotting system (2).

In the years since this discovery, we and others have identified a variety of procoagulant and proinflammatory roles for polyP (11). In particular, we identified four points in the clotting cascade that are modulated by polyP (always in a procoagulant manner), and furthermore, we found that the polymer length of polyP has a profound impact on its ability to support coagulation reactions (11, 12). Thus, long-chain polyP, such as is found in microorganisms (5), reported to be present in mammalian brain (6), and released by prostate cancer cells (9), is an especially potent activator of the contact pathway (12). Shorter-chain polyP such as is secreted by activated platelets, mast cells, and basophils (8, 10, 13), only weakly activates the contact pathway (12). On the other hand, polyP of the size secreted by platelets potently modulates downstream clotting reactions, including promoting factor XI activation by thrombin (14), promoting factor V activation by multiple proteases (11), enhancing fibrin clot structure and delaying fibrinolysis (2, 15, 16), and abrogating the anticoagulant function of tissue factor pathway inhibitor (2, 12). Perhaps surprisingly, platelet-sized polyP is also a potent inhibitor of the complement cascade (17, 18).

In 2007, the Preissner laboratory reported for the first time that RNA and DNA can trigger the contact pathway of blood clotting, and further showed that intravenous ribonuclease infusion protected animals from experimentally induced thrombosis (3). The possible contributions of extracellular nucleic acids to coagulation and inflammation are now under intense investigation, with a number of studies reporting the ability of cell-free RNA (3) and DNA (19–21) to activate the contact pathway, particularly in the context of release of DNA in neutrophil extracellular traps (NETs) (22–24). PolyP and nucleic acids are both linear polymers with repeating, regularly spaced anionic phosphate groups, so it is perhaps not surprising that they both may support activation of the contact pathway.

In this manuscript, we present some caveats that must be considered when interpreting data describing the *in vitro* procoagulant activity of nucleic acids. This includes reviewing our previously published work describing the potential contamination of purified nucleic acids with procoagulant silica particles, and providing new data comparing the relative procoagulant activities of polyP versus nucleic acids.

## Materials and Methods

### Materials

Polyphosphate was size-fractioned as described (12) to obtain preparations indicated as: short-chain polyP (mode, 75 phosphates; range, 20–330 phosphates); medium-chain polyP (mode, 385 phosphates; range, 265–630 phosphates); and long-chain polyP (mode, 1,195 phosphates; range, 390 to more than 2,000 phosphates).

**Figure 1:**
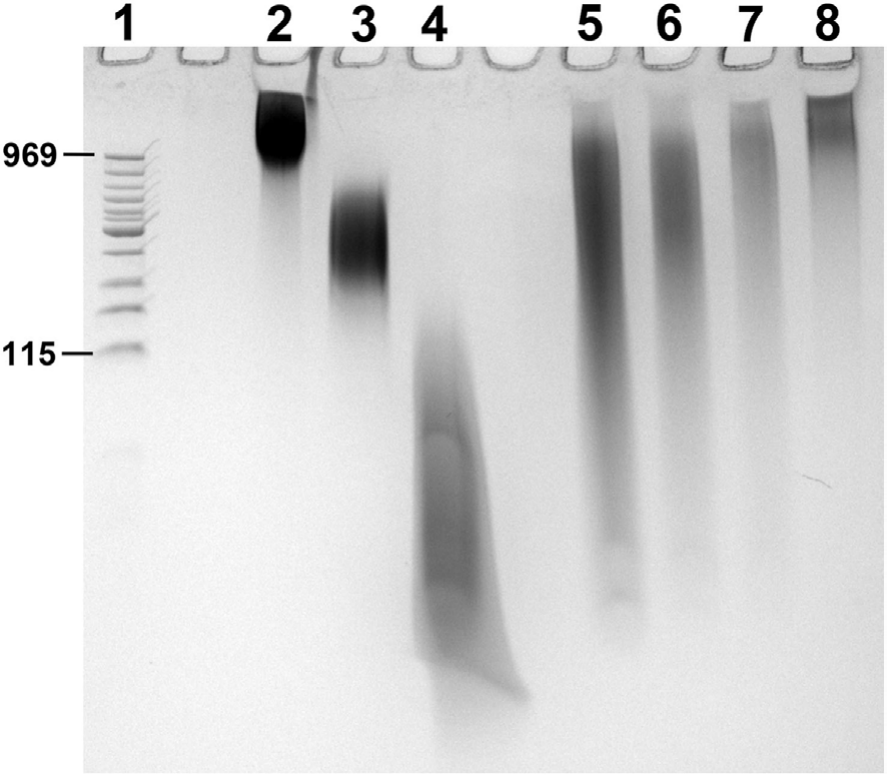
PAGE of RNA homopolymers and polyphosphate (polyP). PolyP preparations and RNA homopolymers used in this study were resolved by PAGE using a TBE-urea gel (10% polyacrylamide). 2 µg of each sample was loaded into the following lanes, with 2 M urea in the loading buffer: (1) low MW DNA ladder (with corresponding polyP sizes marked in terms of phosphate units); (2) long-chain polyP; (3) medium-chain polyP; (4) short-chain polyP; (5) polyI; (6) polyG; (7) polyC; and (8) polyA. PolyP and nucleic acids were stained for 5 min with 0.05% toluidine blue in 5% glycerol/25% methanol, and de-stained overnight in 5% glycerol/25% methanol.

Citrated pooled normal plasma was from George King Biomedical. Phospholipid vesicles were made by sonication using phospholipids from Avanti Polar Lipids (85% 1-palmitoyl-2-oleoyl-*sn*-glycero-3-phosphocholine/15% 1-palmitoyl-2-oleoyl-*sn*-glycero-3-L-serine). Calf intestinal alkaline phosphatase (CIAP) was from Promega and bacteriophage lambda DNA, and DNA ladder were from New England Biolabs. SYBR Green I was from FMC BioProducts. Benzonase, 4′,6-diamidino-2-phenylindole (DAPI), Baker’s yeast tRNA, and RNA homopolymers (polyguanylate, polyG; polyinosinate, polyI; polyadenylate, polyA; or polycytidylate, polyC) were from Sigma-Aldrich. A size comparison of polyP and RNA homopolymers is given in Figure 1.

### Comparison of PolyP Recovery Rates Using Nucleic Acid Purification Kits

DNeasy Blood and Tissue kit (Qiagen), RNeasy Mini kit (Qiagen), Qiaquick PCR Purification kit (Qiagen), TRIzol Reagent (Invitrogen), and phenol/chloroform extraction were evaluated for their recovery rates when used to “purify” samples consisting of nucleic acid alone (either phage lambda DNA or polyG), or nucleic acid mixed with long-chain polyP. We employed 5 µg of each nucleic acid and polyP for such experiments with the phenol/chloroform extraction and the DNeasy, RNeasy, and TRIzol kits, and 2.5 µg of each nucleic acid and polyP for the Qiaquick kit (because of its lower capacity). Instructions for the DNeasy kit vary according to source material, so the protocol for “cultured cells” was followed and the optional second elution of DNA was included. For the RNeasy kit, the protocol was followed for samples with less than 5 × 10^6^ animal cells; the optional membrane-drying spin was included; but the optional on-column DNase digestion was omitted. Sample preparation for TRIzol purification used the protocol for suspension cells. The TRIzol reagent allows for isolation of both RNA and DNA; we performed sequential isolation of nucleic acids and/or polyP from both the aqueous (RNA-containing) and interphase/organic (DNA-containing) layers as described in the manufacturer’s instructions. Nucleic acid concentrations were determined by A_260_. PolyP concentrations were determined following complete digestion with 1 µg/ml recombinant yeast exopolyphosphatase [PPX1, purified as described (25)] in 60 mM Tris-HCl, pH 7.4, and 6 mM MgCl_2_ at 37°C for 30 min, after which the released monophosphate was quantified by PiBlue assay (BioAssay Systems).

### Isolation of Cell-Derived Nucleic Acids

DNA from HEK 293 cells and NETs was prepared *via* phenol/chloroform extraction as described (26). Cellular RNA was purified using RNeasy Midi kit (Qiagen), according to the manufacturer’s instructions. Nucleic acid concentrations were determined by A_260_.

### Evaluation of PolyP Contamination in Cell-Derived DNA

DNA purified from HEK 293 cells using the DNeasy Blood & Tissue kit was digested to hydrolyze the nucleic acids with 700 U/mL benzonase in 20 mM HEPES pH 7.4, 5 mM NaCl, 5 mM KCl for 30 min at 37°C. Any residual polyP was then isolated as described (9) with minor modifications. Briefly, 8 M LiCl and 4.5 M NaI were added to the samples, which were then applied to EconoSpin DNA spin columns (Epoch Life Sciences). Columns were washed five times with a solution of 50% ethanol, 100 mM NaCl, 10 mM Tris pH 7.5, 1 mM EDTA, after which polyP was eluted in purified water. The polyP was then digested with PPX1 and the released monophosphate quantified as described above. Some of the resulting material was then subjected to digestion with 300 U/mL CIAP in 20 mM HEPES pH 7.4, 5 mM NaCl, 5 mM KCl for 30 min at 37°C. HEK 293 cell DNA digested with benzonase alone, or benzonase followed by CIAP, was subjected to PAGE on 4–20% gels. The gels were visualized with DAPI staining for polyP (negative fluorescent staining after photobleaching) as described (27), or SYBR Green I for nucleic acid.

### Contact Pathway-Initiated Plasma Clotting Assays

Plasma clot times were quantified in duplicate at 37°C by mixing 50 µL of activator (nucleic acid or polyP in 20 mM HEPES pH 7.4, 5 mM NaCl, 5 mM KCl with 75 µM phospholipid vesicles) and 50 µL of plasma in wells of medium-binding 96-well polystyrene microplates (Corning). After 3 min, 50 µL of pre-warmed 25 mM CaCl_2_ in 20 mM HEPES was added and mixed in by trituration. Light scattering (A_405_) was monitored in a SpectraMax 340PC microplate reader (molecular devices) at 37°C. Clot times were determined as previously described (28).

## Results and Discussion

### First Caveat: Silica Particles Contribute to the Procoagulant Activity of DNA, RNA, or PolyP Isolated Using Some Commercial Kits

In this section, we review and discuss the implications of our recently published study (26). Our initial goal in that study was to perform a direct, head-to-head comparison of the procoagulant activity of polyP versus nucleic acids isolated from various cellular sources. To carry out this comparison, we isolated DNA from human cells using Qiagen kits, since these kits had been employed in multiple published studies of the procoagulant activity of DNA (3, 20, 21, 23, 29–31) and RNA (3, 30). These kits, which are convenient and rapid, exploit the anionic nature of DNA and RNA by selectively adsorbing them onto a silica matrix contained in spin columns. Binding of nucleic acids to silica occurs only under high-salt conditions, and after washing, the nucleic acids are eluted using low-salt conditions (32). Notably, some recent studies have also used such Qiagen kits to isolate polyP from human platelets and prostate cancer cells, in order to study the procoagulant activity of polyP from these cellular sources (9, 33, 34).

When we used such Qiagen kits to isolate DNA from human cells, we found that the resulting DNA preparations exhibited marked batch-to-batch variation in ability to trigger plasma clotting (26). In sharp contrast, DNA purified from the same cellular sources using traditional phenol–chloroform extraction exhibited much less procoagulant activity, and with less batch-to-batch variability. This result suggested that the Qiagen kit purification technique somehow increased the procoagulant nature of the purified DNA. We further demonstrated that materials with minimal to no procoagulant activity (such as purified bacteriophage lambda DNA or short-chain polyP) acquired substantial procoagulant activity when re-purified using these silica columns. Additionally, mock purifications using phosphate-buffered saline without cells, or just washing the spin columns with water, resulted in eluates with readily quantifiable procoagulant activity. The procoagulant activity of such eluates was also observed when we used silica-based spin columns from sources other than Qiagen, and we found that this clotting activity was highly variable. These experiments supported the notion that the spin columns in these kits release a procoagulant substance into the eluted material.

Silica is potently procoagulant (35), and in fact, colloidal silica is the clot-triggering substance in several commercially available aPTT reagents. In our study (26), we confirmed the procoagulant activity of silica particles using a preparation of acid-washed silica particles commonly termed “glass milk.” When we removed the matrix from Qiagen spin columns, we homogenized it and added it to clotting assays, we found that it was as procoagulant as glass milk. We, therefore, hypothesized that Qiagen spin columns leach variable amounts of silica particles that contribute to the observed procoagulant activity of any DNA, RNA, or polyP purified using such kits. We found that much of the procoagulant activity of DNA purified on such silica spin columns survived either nuclease digestion or boiling in 1 N HCl—as did all the clotting activity of glass milk (26). Elemental analysis of eluates from Qiagen spin columns confirmed the presence of silicon in quantities consistent with the observed clotting activities. These findings all supported the conclusion that spin columns in popular kits for purifying nucleic acids release silica particles in amounts that exhibit readily measurable clotting activity. We attempted to remove this procoagulant material from kit-purified DNA using a variety of filtration, centrifugation, and precipitation approaches, but were unable to identify conditions that did not also result in loss of large amounts of the DNA (data not shown). This suggests that a subset of the silica particles are so small that they cannot be easily removed using either filtration or centrifugation.

The main conclusion from our previous study (26) was that DNA, RNA, or polyP that are purified using silica-based approaches, such as Qiagen kits, will be contaminated with variable amounts of procoagulant silica particles that cannot easily be removed. These kits were designed for purification of materials for molecular biology applications, and should not be used to purify materials when contaminant silica will impact the experimental results in downstream applications, such as plasma clotting assays. This has implications for the conclusions of some recently published studies. For example, Noubouossie et al. (31) reported that neutrophil NETs were not procoagulant, but that DNA purified from such NETs was procoagulant. When they added histones back to the isolated DNA to reconstitute a chromatin-like state, the material lost its procoagulant activity. However, that study employed Qiagen kits to isolate DNA from NETs, so the observed procoagulant activity of this DNA may reflect a contribution from silica particles. Furthermore, we found that histones at concentrations employed in that study (31) can passivate silica particles and mask their procoagulant activity (26). This could, therefore, be an alternative explanation for why adding histones to Qiagen kit-purified DNA rendered the material unable to trigger the contact pathway of clotting.

In a separate study, Verhoef et al. (34) isolated polyP from human platelets using both phenol/chloroform extraction and Qiagen PCR purification kits. They reported that phenol/chloroform-extracted polyP had little ability to trigger the contact pathway, while polyP isolated using the Qiagen kits was highly procoagulant. Their conclusion was that the Qiagen kits isolated long-chain polyP from platelets that was not extracted by phenol/chloroform. An alternative explanation is that the Qiagen kit-purified polyP may have contained highly procoagulant silica particles.

### Second Caveat: Nucleic Acids and PolyP Co-Purify

DNA, RNA, and polyP have similar physical properties as they are all anionic polymers containing regularly spaced phosphates. This explains why they can be isolated from biological samples using similar methods such as phenol/chloroform extraction or silica-based spin columns. Since many methods developed for purifying nucleic acids have also been used to isolate polyP, we were interested to determine if polyP and nucleic acids might co-purify.

**Figure 2:**
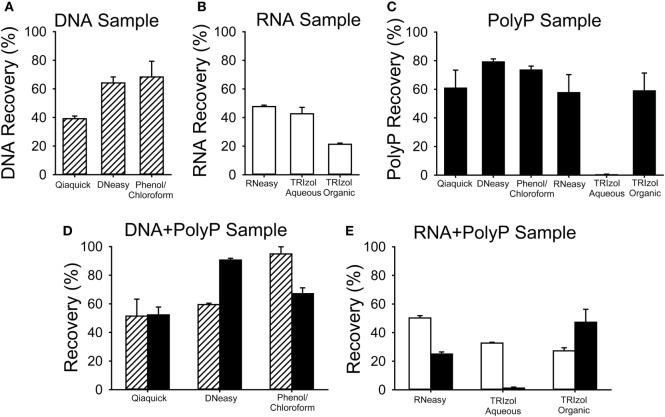
Recovery of nucleic acids and polyphosphate (polyP) using various purification methods. Known quantities of bacteriophage lambda DNA (DNA, striped bars), polyG (RNA, white bars), or long-chain polyP (black bars) were processed for nucleic acid purification using Qiaquick, DNeasy, or TRIzol kits, following the manufacturer’s instructions, or with phenol/chloroform extraction. Nucleic acid recovery was quantified using A_260_ and polyP recovery by digestion with PPX1 and PiBlue assay. Results are plotted as percent of the input amount in each experiment. For all experiments except the Qiaquick kits, the three samples were: **(A)** DNA alone (5 µg lambda DNA); **(B)** RNA alone (5 µg polyG); **(C)** 5 µg polyP alone; **(D)** a mixture of 5 µg polyP plus 5 µg of DNA; or **(E)** 5 µg polyP plus 5 µg of RNA **(E)**. Experiments with the Qiaquick kit employed 2.5 µg of polyP and/or nucleic acid. Data are mean ± SEM (*n* = 3).

To examine this question, we quantified the recovery of nucleic acids and polyP using a variety of popular methods for isolating DNA or RNA from cells or tissues (Figure 2). In these experiments, known quantities of purified bacteriophage lambda DNA or a synthetic RNA homopolymer, polyG, either alone or mixed with long-chain polyP, were purified using phenol/chloroform extraction or Qiaquick kits (Qiagen), DNeasy kits (Qiagen), RNeasy Miniprep kits (Qiagen), or TRIzol kits (Thermo Fisher), following the manufacturers’ instructions. Purification of DNA with the Qiaquick kit, the DNeasy kit, or with phenol/chloroform resulted in 40–60% recovery of the applied DNA (Figure 2A), and adding polyP to the sample did not diminish the DNA recovery (Figure 2D). Purification of RNA with the RNeasy Miniprep kit resulted in ~50% RNA recovery, regardless of whether polyP was present (Figures 2B,E). Similarly, the presence of polyP did not diminish RNA recovery from either the aqueous or organic phase when using TRIzol reagent (Figure 2E).

When polyP was present in the starting nucleic acid sample, it was readily recovered alongside the DNA or RNA using all the kits tested. The Qiaquick kit yielded ~55% of the starting polyP, when purified alone (Figure 2C) or in the presence of DNA (Figure 2D). With the DNeasy kit, polyP recovery was higher (91%) when DNA was present in the sample (Figure 2D) than when DNA was absent (79%) (Figure 2C). PolyP (58%) was also recovered with the RNeasy kit (Figure 2C). Little or no polyP was recovered in the aqueous phase of TRIzol (which is typically used to isolate RNA), while 50–60% of polyP was recovered from the interphase/organic phase (which typically contains DNA).

**Figure 3:**
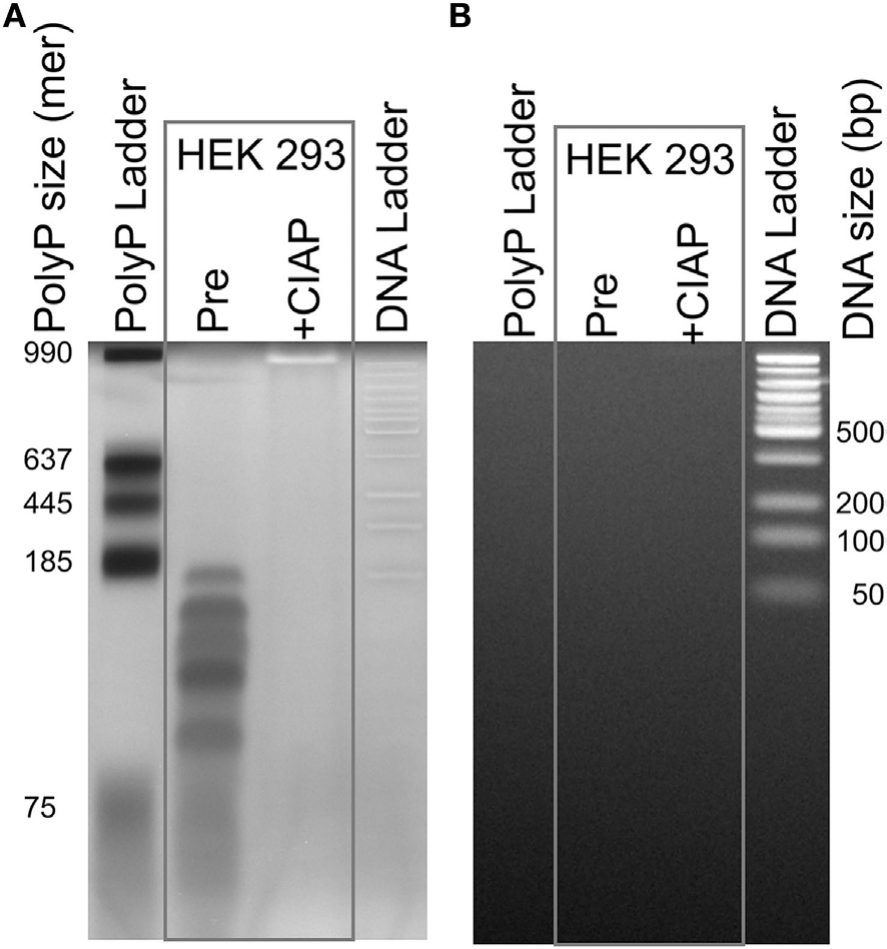
Traces of polyphosphate (polyP) in cell-derived DNA. DNA isolated from HEK 293 cells using the DNeasy Blood & Tissue kit was extensively digested with benzonase to hydrolyze the DNA, concentrated, and then resolved by electrophoresis on a 4–20% polyacrylamide gel. Samples were: a locally prepared polyP ladder (lengths indicated in phosphate units); DNA purified from HEK 293 cells and digested with benzonase (“Pre”); the same material following digestion with calf intestinal alkaline phosphatase (CIAP); and 50 bp DNA ladder. The same gel was stained sequentially, using: **(A)** DAPI with extended photobleaching to detect polyP (27) and **(B)** SYBR Green I to detect DNA (after removing DAPI by repeated rinsing). The material in the lane marked “Pre” is clearly polyP as it photobleached rapidly **(A)**, was digested by CIAP **(A)**, and did not stain with SYBR Green 1 **(B)**.

To visualize trace levels of polyP contamination in HEK 293 cell DNA, we purified 500 µg of DNA from these cells using the DNeasy kit, extensively digested the DNA with benzonase (a highly active, nonspecific nuclease), and then resolved the resulting preparation using polyacrylamide gel electrophoresis. We then stained the gels sequentially with DAPI followed by SYBR Green I (Figure 3). DAPI fluoresces when bound to either nucleic acids or polyP, but DAPI-DNA photobleaches very slowly with exposure to UV light, while DAPI-polyP photobleaches extremely rapidly (27). SYBR Green I fluorescently stains DNA but not polyP. When the sample purified from HEK 293 cells was stained with DAPI, we readily observed material that photobleached (DAPI negative stain; Figure 3A), but did not stain with SYBR Green I (Figure 3B). SYBR Green 1 staining confirmed that benzonase digestion completely degraded the HEK 293 cell DNA (Figure 3B), which is consistent with the residual material being polyP, not nucleic acid. Further, CIAP [a highly active exopolyphosphatase (36)] digestion of this material completely abrogated DAPI negative staining, confirming the presence of polyP. PolyP from HEK 293 cells was predominately short-to-medium length, with the longest polymers less than 200 phosphate units in length. We recovered 1.9 nmol of polyP-derived monophosphate from a DNA sample containing 1.23 μmoles of nucleotides (assuming an average nucleotide molecular weight of 327 Da). Thus, the polyP content in this sample, on a molar basis, was 0.16% that of DNA (i.e., moles of phosphate in polyP relative to moles of nucleotide in DNA).

Collectively, these data demonstrate that polyP can readily co-purify with nucleic acids using popular methods. Since polyP is ubiquitous in biology and is found in many mammalian cells and tissues (4, 6), it seems likely that DNA or RNA isolated from cells or tissues will be contaminated with whatever polyP was also present. If so, then the procoagulant activity of such DNA or RNA preparations may be the sum of the clotting activities of the nucleic acids plus that of any contaminating polyP. This problem can be addressed treating nucleic acid preparations with CIAP, which hydrolyzes polyP, but not DNA or RNA. This step is recommended in future studies of the clotting activity of nucleic acids.

### Long-Chain polyP Is Orders of Magnitude More Procoagulant Than Nucleic Acids

**Figure 4:**
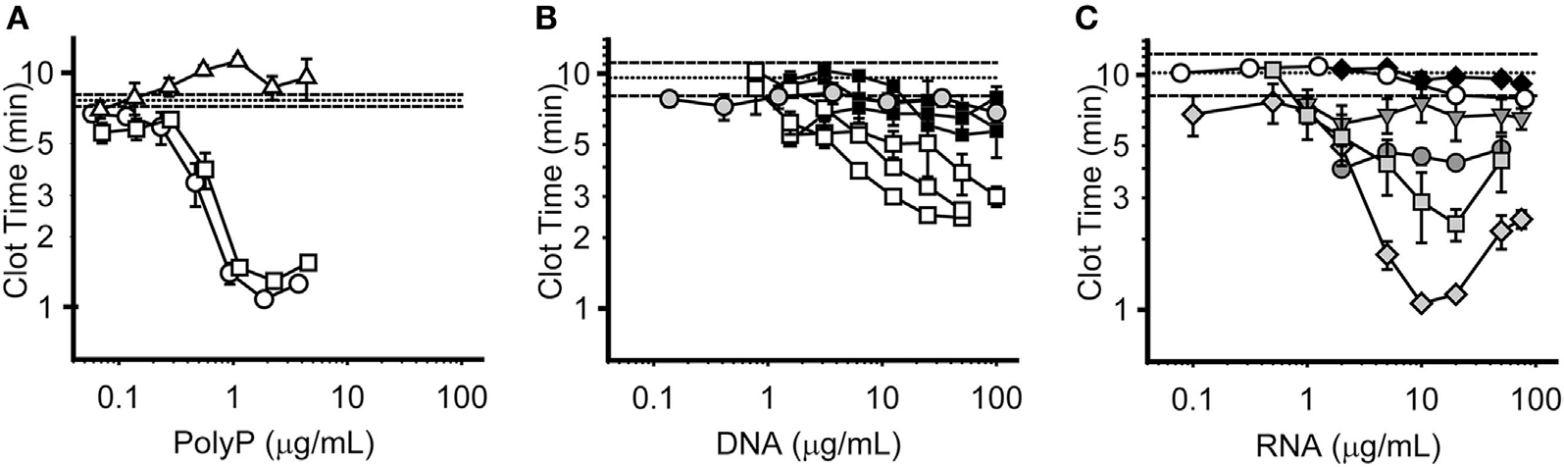
Procoagulant activities of polyphosphate (polyP), DNA, and RNA. Panels display plasma clot times versus concentration, with horizontal dashed lines showing the mean clot time without activator (±SEM as horizontal dotted lines). Symbols represent the mean, and error bars the SE, of three separate experiments evaluating clot times versus concentration. **(A)** Clot times with short-chain polyP (74 mer, Δ); medium-chain polyP (385 mer, □); and long-chain polyP (1195 mer, ○). **(B)** Clot times with HEK 293 cell DNA isolated with phenol/chloroform (□, 3 distinct purifications); NET-derived DNA isolated with phenol/chloroform (■, 3 distinct purifications); or lambda DNA (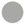). Note that for comparison purposes, the clotting data for HEK 293 DNA and NET DNA in this panel are replotted from those in Supplemental Figure S2 in Supplementary Material of Smith et al. (26). **(C)** Clot times with HEK 293 cell RNA (◆); baker’s yeast transfer RNA (○); or the RNA homopolymers, polyA (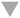), polyC (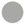), polyI (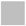), or polyG (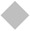). PolyU was also tested, but had no procoagulant activity (data not shown).

We compared the relative procoagulant activities of nucleic acids versus polyP. To do so, we utilized a modified aPTT clotting assay in which varying concentrations of polyP, RNA, or DNA were added to plasma to trigger clotting *via* the contact pathway. We previously reported (12), that polyP shortened the time to clot formation in a manner that was dependent on both concentration and polymer length. Short-chain polyP of the size released from platelets (approximately 75 units in length) had little ability to trigger clotting *via* the contact pathway, while medium- and long-chain polyP were highly procoagulant, exhibiting maximal shortening of the clot time by 83 and 89%, respectively, at 2 µg/mL polyP (Figure 4A). These results are consistent with our previous data describing the size dependence of polyP-mediated activation of the contact pathway (12). In contrast, both mammalian cell-derived DNA (purified from either HEK 293 cells or NETs using phenol/chloroform extraction) and bacteriophage lambda DNA were weaker than polyP in triggering plasma clotting, and less potent on both a mass and a molar basis (Figure 4B). For HEK 293 cell-derived DNA, maximal shortening of clot time was 73% at 50 µg/mL. For DNA derived from human NETs, maximal shortening was 93% at 50 µg/mL, although DNA preparations derived from different donor cells demonstrated variable procoagulant activity. For lambda DNA, maximal shortening was 29% at 100 µg/mL. HEK 293 cell-derived RNA and yeast tRNA were even less effective in triggering clotting, as they did not appreciably shorten the clot time even at concentrations up to 100 µg/mL (Figure 4C). Interestingly, some RNA homopolymers—in particular, polyG and polyI—were significantly procoagulant, although maximal activity required relatively high concentrations (10–20 µg/mL).

## Summary

In conclusion, methods such as Qiagen kits that employ silica for isolating nucleic acids or polyP are likely to result in contamination of the eluted material with variable amounts of highly procoagulant silica particles. The presence of such silica particles can confound the interpretation of plasma clotting assays conducted with such materials. Furthermore, we show that polyP can readily co-purify with DNA or RNA when utilizing traditional purification methods for nucleic acids. The possible presence of polyP in nucleic acid preparations is, therefore, an additional potentially confounding variable when conducting plasma clotting tests with DNA or RNA, unless steps are taken to either remove or hydrolyze any contaminating polyP. For assessing the procoagulant activity of cellular DNA or RNA, we, therefore, recommend that such nucleic acids should be purified using a method that avoids silica or other matrices that might shed procoagulant particles, and that any contaminating polyP be hydrolyzed by CIAP digestion.

## Author Contributions

JG and SS designed and performed experiments, analyzed results, and edited the manuscript. JM designed experiments, analyzed results, and edited the manuscript.

## Conflict of Interest Statement

JG is employed by L.E.K. Consulting. JM and SS are co-inventors on patents and pending patent applications related to medical uses of polyP.
